# The mTOR–Autophagy Axis and the Control of Metabolism

**DOI:** 10.3389/fcell.2021.655731

**Published:** 2021-07-01

**Authors:** Nerea Deleyto-Seldas, Alejo Efeyan

**Affiliations:** Metabolism and Cell Signaling Laboratory, Spanish National Cancer Research Center (CNIO), Madrid, Spain

**Keywords:** autophagy, mechanistic target of rapamycin, lysosome, metabolism, nutrients

## Abstract

The mechanistic target of rapamycin (mTOR), master regulator of cellular metabolism, exists in two distinct complexes: mTOR complex 1 and mTOR complex 2 (mTORC1 and 2). MTORC1 is a master switch for most energetically onerous processes in the cell, driving cell growth and building cellular biomass in instances of nutrient sufficiency, and conversely, allowing autophagic recycling of cellular components upon nutrient limitation. The means by which the mTOR kinase blocks autophagy include direct inhibition of the early steps of the process, and the control of the lysosomal degradative capacity of the cell by inhibiting the transactivation of genes encoding structural, regulatory, and catalytic factors. Upon inhibition of mTOR, autophagic recycling of cellular components results in the reactivation of mTORC1; thus, autophagy lies both downstream and upstream of mTOR. The functional relationship between the mTOR pathway and autophagy involves complex regulatory loops that are significantly deciphered at the cellular level, but incompletely understood at the physiological level. Nevertheless, genetic evidence stemming from the use of engineered strains of mice has provided significant insight into the overlapping and complementary metabolic effects that physiological autophagy and the control of mTOR activity exert during fasting and nutrient overload.

## The mTOR–Autophagy Axis

### mTOR, Master Regulator of Metabolism

The mechanistic target of rapamycin (mTOR; also referred to as mammalian target of rapamycin) is an evolutionarily conserved kinase and the catalytic core of two distinct complexes: mTOR complex 1 and 2 (mTORC1 and mTORC2) defined by the presence of the key accessory proteins Raptor and Rictor, respectively. These two distinct complexes differ in substrate specificity, in their upstream regulatory cues, and in their subcellular localization. As part of mTORC1, mTOR drives most anabolic processes in the cell, including protein, lipid, cholesterol, and nucleotide synthesis, while it simultaneously boosts extracellular nutrient uptake and blocks autophagic catabolism (Buttgereit and Brand, 1995; Saxton and Sabatini, 2017; Valvezan and Manning, 2019). The coordination of such energetically onerous anabolic programs is coupled to (1) the availability of cellular nutrients and (2) the signals from the organismal nutritional state in the form of second messengers such as insulin (Efeyan et al., 2015; Shimobayashi and Hall, 2016).

### Nutrients, Growth Factors, and mTORC1

**FIGURE 1:**
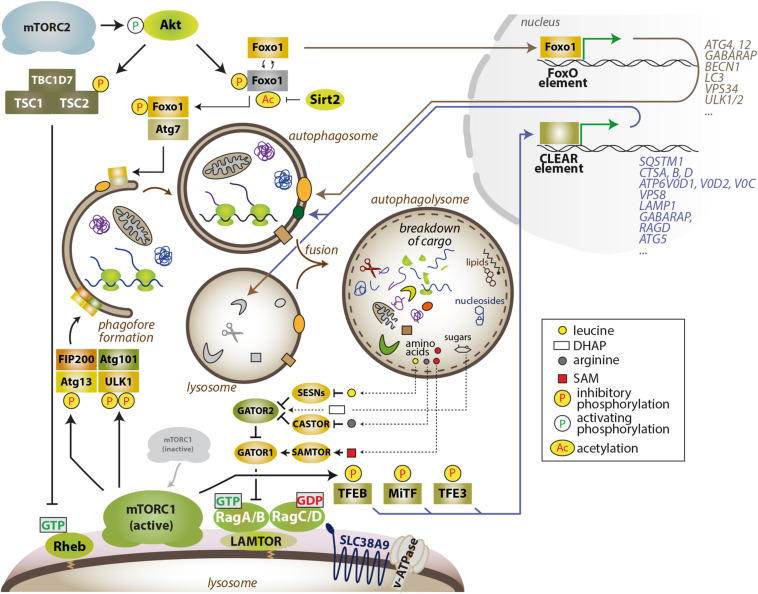
The mTOR–autophagy axis. MTORC1 blocks the early steps in autophagy by phosphorylation-dependent inhibition of Atg13 and ULK1 and also restrains the degradative capacity of the cell by inhibiting the activity of TFEB family members. Several autophagy-related proteins and other lysosomal factors are encoded by genes that harbor a CLEAR sequence in their promoter region, and are bound and transactivated by active TFEB family members. Thus, inhibition of mTORC1 enables autophagic degradation and degradative capacity of the cell, while also suppressing anabolism and, thus, lowering the demand for energy and nutrients. In turn, lysosome-derived amino acids (leucine and arginine) and also other nutrients (glucose-DHAP, cholesterol, SAM) can partially reactivate Rag GTPase – nutrient signaling upstream of mTORC1. MTORC2 and the AGC kinases Akt and SGK1 also limit the autophagic flux and lysosomal capacity via transcription-dependent and -independent processes. The cytoplasmic sequestration of the FoxO transcription factors indirectly limits autophagic degradation, as several FoxO targets encode proteins that directly participate in different steps of autophagy and lysosomal catabolism.

The signal transduction cascade from hormones/growth factors that activates the mTOR kinase starts with the activation of a receptor tyrosine kinase at the plasma membrane that switches on the PI3K–Akt axis, which results in the inhibition of the tuberous sclerosis complex (TSC). TSC integrates inputs from cellular stress, such as hypoxia and limiting ATP levels, and is a GTPase-activating protein for the small GTPase Rheb (Garami et al., 2003; Inoki et al., 2003; Tee et al., 2003; Zhang et al., 2003). When bound to GTP, Rheb induces a conformational change in mTORC1 that results in kinase activation (Anandapadamanaban et al., 2019; Rogala et al., 2019). Importantly, Rheb is anchored at the outer surface of the lysosome, and it can only interact with and activate mTORC1 if mTORC1 is tethered to the outer lysosomal surface, a re-localization process that occurs in a cellular nutrient-dependent manner (Sancak et al., 2008; Figure 1).

The levels of cellular nutrients (amino acids, glucose, certain lipids, and likely other metabolites) are sensed and signaled by an expanding number of proteins (SESNs, CASTOR, KICSTOR, SAMTOR, GATOR1 and 2) (Saxton and Sabatini, 2017) that culminate in the control of the guanosine phosphate state of the members of the Rag family of GTPases, which bind mTORC1 in a nutrient-sensitive manner (Figure 1). In addition, Rag GTPase-independent amino acid control of mTORC1 exists (Efeyan et al., 2014; Jewell et al., 2015). The Rag GTPases operate as obligate heterodimeric partners composed of RagA or RagB plus RagC or RagD (Schurmann et al., 1995). In the presence of cellular nutrients, RagA or B are loaded with GTP, while RagC or D are loaded with GDP (Kim et al., 2008; Sancak et al., 2008). This specific nucleotide configuration allows the interaction with mTORC1 and its recruitment to the outer lysosomal surface, where the Rheb–mTORC1 interaction, and mTORC1 activation, occurs. Hence, the current paradigm of the regulation of mTORC1 states that maximal mTORC1 activity takes place when both signals are present: (1) cellular nutrients to recruit mTORC1 and (2) hormones/growth factors for kinase activation (Lawrence and Zoncu, 2019; Valvezan and Manning, 2019). This elegant mechanism of coincidence detection of inputs has been well illustrated by biochemical, cell biological, and structural approaches, but we still ignore how the convergent controls of mTORC1 activity result in the execution of a coherent, multifaceted metabolic program in real organs with physiological and pathological fluctuations in nutrients and hormones.

### mTORC1, Catabolism, and Biosynthetic Capacity

At the cellular level, intense research in the last 15 years has shown that the anabolic program executed by mTORC1 is coupled to a block in catabolic processes. Otherwise, a disconnection would result in futile, cyclic synthesis and degradation of cellular components and biomass. Nonetheless, the increased demand for energy and building blocks for macromolecule synthesis upon mTORC1 activation, together with the block in autophagy, results in a biosynthetic burden that the cell alleviates with the execution of parallel programs that reinforce nutrient uptake (Park et al., 2017; Torrence et al., 2020) and synthesis (Robitaille et al., 2013; Ben-Sahra et al., 2016; Valvezan et al., 2017), and also enables the catabolic capacity of the cells through the proteasomal degradation of proteins (Zhang et al., 2014) to remove unwanted cellular material and to boost the pool of free amino acids available for protein synthesis. Reciprocally, an increased autophagic flux and the recycling of cellular macromolecules upon extended inhibition of mTORC1 is sufficient to partially replenish intracellular or intra-lysosomal amino acid pools and to partially reactivate mTORC1 (Yu et al., 2010; Figure 1).

### mTORC1 Governs Autophagy at the Lysosomal Surface

It is not coincidental that the subcellular location where mTORC1 activation occurs is the cytoplasmic side of the organelle responsible for internal recycling of macromolecules: the lysosome. Indeed, such spatial association has remained fixed throughout eukaryotic and metazoan evolution. The cytoplasmic side of the lysosomal membrane provides a scaffold surface for controlling mTORC1 and also enables immediate control of specific mTORC1 targets associated to the lysosome (Rabanal-Ruiz and Korolchuk, 2018). Moreover, both abundance and positioning of lysosomes are important modulators of mTORC1 and mTORC2 (Korolchuk et al., 2011; Jia and Bonifacino, 2019; Mutvei et al., 2020). Targets of mTORC1 that are transiently or permanently associated to the lysosome include ULK1, ATG13, and TFEB and its family members, but exactly how and where mTORC1 reaches and phosphorylates its targets is not clear. The coordinated control of these targets results in complementary functions that, together, tightly restrict autophagy when mTORC1 is active. On one side, the inhibitory phosphorylation of the ULK1/ATG13/FIP200 complex by mTORC1 immediately blocks the initiation of the formation of the autophagosome (Ganley et al., 2009; Hosokawa et al., 2009; Kim et al., 2011). This early block is reinforced by the inhibitory phosphorylation of TFEB and MiTF-TFE family members (Settembre et al., 2011; Roczniak-Ferguson et al., 2012; Martina and Puertollano, 2013). TFEB and family members are themselves a signaling hub that computes information from nutrient sufficiency, calcium signaling (Medina et al., 2015), and overall lysosomal stress and health (Ballabio and Bonifacino, 2020), and ultimately execute a transcriptional program that includes the synthesis of proteins that boost the degradative capacity of the cell (Napolitano and Ballabio, 2016; Ballabio and Bonifacino, 2020). The lysosomal biogenesis program encompasses lysosomal membrane proteins, lysosomal lumen enzymes that execute the catalytic degradation of cargo, and factors directly involved in the execution of autophagy (Sardiello et al., 2009; Settembre et al., 2011; Figure 1). Thus, the coordinated actions of mTORC1 ensure a rapid block in autophagy and a slower inhibition of this transcriptional program that ultimately restricts lysosomal biogenesis and lysosomal function (Figure 1).

As mentioned, phosphorylation of mTORC1 targets results in induction and in inhibition of anabolic and catabolic functions, respectively. A consensus sequence that facilitates recruitment to, and phosphorylation by mTORC1 has been inceasingly refined (Hsu et al., 2011; Yu et al., 2011; Robitaille et al., 2013). While for years the paradigm has stated that all mTORC1 targets interact with mTORC1 by means of the TOR signaling (TOS) motif (Schalm and Blenis, 2002), recent compelling work has demonstrated that this is not universal. In particular, TFEB family members are orphan of a TOS motif and are instead recruited to mTORC1 through a direct interaction with the RagC GTPase (Napolitano et al., 2020). Importantly, inhibition of TFEB by mTORC1 is facilitated by RagC exclusively under cellular nutrient sufficiency, thus defining an input-dependent asymmetry in the activation of different targets downstream of mTORC1 (Napolitano et al., 2020). While targets with a TOS motif result phosphorylated only if the TSC-Rheb and the Rag GTPase arms are *ON*, TFEB is sensitive to any perturbation that would result in a change in the nucleotide to which RagC is bound, such as nutrient withdrawal/replenishment, but would be less affected by the inhibition and activation of the growth factor signaling cascade.

The physiological implications of this asymmetric control of mTORC1 targets are enormous and have been already underlined in the context of brown adipose tissue function (Wada et al., 2016), and of the Birt–Hogg–Dubé syndrome (Napolitano et al., 2020), and may underlie the occurrence of activating mutations in RagC (but not in RagA) in B-cell lymphomas (Ortega-Molina et al., 2019).

### The Regulation of Autophagy by mTORC2

In addition to the suppression of autophagy when part of mTORC1, the mTOR kinase restrains autophagy when part of mTORC2. Through mechanisms that are not entirely defined, growth factor signaling activates mTORC2, and upon its activation, mTORC2 phosphorylates and activates Akt and other members of the AGC kinase family (Hoxhaj and Manning, 2020). The FoxO transcription factors are phosphorylation targets of Akt (Brunet et al., 1999), and Akt-dependent phosphorylation of FoxO1/3a results innuclear exclusion, thus impairing the transactivation of autophagy-related factors and lysosome-tethered proteins (Mammucari et al., 2007; Zhao et al., 2007). In an interesting crosstalk between Akt and the NAD-dependent deacetylase family of Sirtuins, Sirt1 also modulates the transcriptional activity of FoxO1 (Frescas et al., 2005). Moreover, in an apparently counterintuitive manner, cytoplasmic FoxO1 can also induce the autophagic flux by its interaction with Atg7 (Zhao et al., 2010), and this cytoplasmic, transcription-independent function of FoxO1 requires its deacetylation by Sirt2, and is facilitated by nuclear exclusion of FoxO1 by Akt-dependent phosphorylation (Zhou et al., 2012). While this nucleo-cytoplasmic tug of war of FoxO1 shuttle in the control of autophagy deserves further investigation, the acetylation of FoxO1 in the cytoplasm ensures the rapid execution of autophagy even in conditions of high growth factor signaling (Figure 1). In addition, not all reported connections between mTORC2 and autophagy involve cytoplasmic and nuclear functions of FoxO, as Akt directly phosphorylates and inhibits ULK1 (Bach et al., 2011). Finally, transcriptional activity by FoxO includes the upregulation of Sestrins, negative regulators of the Rag-mTORC1 axis, in an additional loop of cross-regulation (Chen et al., 2010).

In addition to the functions of Akt, inhibition of another member of the AGC kinase family, SGK1, by either genetic and pharmacological means, induces autophagy *in vitro* and *in vivo* (Conza et al., 2017; Liu et al., 2018; Zuleger et al., 2018), and these effects may also be mediated by the phosphorylation of FoxO3a. In worms, suppression of the mTORC2–SGK1 axis induces autophagy in a DAF-16/FOXO-independent manner (Aspernig et al., 2019) downstream of altered mitochondrial permeability (Zhou et al., 2019), strongly suggesting that mTORC2 lies upstream of the control of autophagy by SGK1. Such control is prominent in mammalian skeletal muscle (Andres-Mateos et al., 2013; Zuleger et al., 2018). However, the fact that SGK1 knock-out mice (Wulff et al., 2002; Fejes-Tóth et al., 2008) show minimal phenotypic alterations, unlike autophagy-deficient mice (Kuma et al., 2004), may reflect the importance of SGK1 under specific stress conditions.

The enumerated multilayered, convergent, and complementary effects of mTORC1 and mTORC2 in the repression of autophagy deciphered at the molecular level point to (1) a direct, acute blockade of the phagophore–autophagosome formation, together with (2) a long-term, transcription-based limitation of the degradative machinery, but how is this complexity integrated to sustain metabolic homeostasis and how may its deregulation contribute to metabolic disease are far less understood. We next review the lessons that mouse genetic approaches have taught in the understanding of the physiology of the mTOR–autophagy axis.

## The mTORC1–Autophagy Axis in Mammalian Metabolism

### mTOR, Autophagy and Mammalian Adaptation to Fasting

Suppression of mTORC1 activity has proven essential to endure conditions of nutrient deprivation in mammals. Immediately after birth, glucose and amino acid levels in circulation drop dramatically due to the interruption of the trans-placental supply, and mTORC1 in mouse tissues is readily inhibited within 1 h after birth. This rapid and strong inhibition is critical to unleash autophagy, which in turn boosts free amino acid levels that feed gluconeogenesis to sustain glycaemia (Efeyan et al., 2013), and is key to endure the first hours of life, before significant maternal milk supply occurs. Neonatal mice carrying a point mutation in *RagA* (RagA^Q66L^, referred to as RagA^GTP^) that renders mTORC1 constitutively active regardless of nutrient levels are unable to trigger autophagy after birth, suffer a profound hypoglycemic state they do not recover from, and finally succumb within 1 day after birth (Efeyan et al., 2013). A strikingly similar phenomenon was observed in mice lacking *Sestrin 1, 2* and *3*, negative regulators of the Rag GTPases (Peng et al., 2014), and in *Atg5*^–/–^ (Kuma et al., 2004) and *Atg7*^–/–^ mice (Komatsu et al., 2005). The phenotypic resemblance of mice with constitutive nutrient signaling and autophagy-deficient mice provides strong genetic and physiological support for the importance of the regulation of autophagy by the nutrient–mTORC1 pathway in the maintenance of metabolic homeostasis (see Table 1).

**TABLE 1:** Metabolic phenotypes of genetically-engineered mice with increased and decreased mTORC1 activity and enhanced or suppressed autophagy.

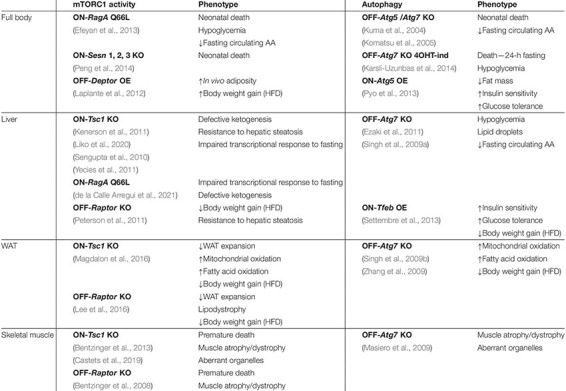

*KO, knock-out; OE, overexpressor; AA, amino acids; 4OHT-ind, tamoxifen-inducible; HFD, high-fat diet; WAT, white adipose tissue.*

The sequential steps in the transition from postprandial to early and long-term fasting metabolism in adult individuals encompass a shift from glucose to lipid and ketone body metabolism, through consistent and complementary responses in the liver, skeletal muscle, and white adipose tissue (WAT), coordinated systemically by hormonal signaling. An early fasting switch involves the release of liver glycogen stores as soon as insulin levels drop. When glycogen has been entirely mobilized, gluconeogenesis occurs together with increased lipolysis from the WAT to yield circulating glycerol, a substrate for gluconeogenesis, and free fatty acids (FFA). The ATP produced by β-oxidation of FFA in the liver sustains gluconeogenesis, while acetyl-CoA is shunted to the production of ketone bodies, an essential source of energy during fasting, and particularly important for the brain.

In addition to its essentiality in neonatal mice, autophagy is required by adult mice to endure periods of fasting. Acute, full-body ablation of autophagy by tamoxifen-mediated deletion of *Atg7* impairs the survival of the mice following a 24-h fast due to severe hypoglycemia. Interestingly, amino acid levels in circulation are sustained by accelerated muscle wasting, but this increase fails to support gluconeogenesis, and decreased WAT and rapidly depleted hepatic glycogen stores compromise the endurance to long-term fasting (Karsli-Uzunbas et al., 2014).

As soon as insulin drops, autophagy is induced in the liver, and adult mice unable to trigger hepatic autophagy fail to release free amino acids and suffer from reduced blood glucose levels during fasting, an effect that can be rescued experimentally by exogenous administration of the gluconeogenic amino acid serine (Ezaki et al., 2011). It is noteworthy that in these mice, glycogen mobilization during fasting is not impaired, and neither is it in *RagA*^GTP^ neonates (Efeyan et al., 2013) nor in adult *Atg7*-KO mice (Karsli-Uzunbas et al., 2014), in spite of the relevance of autophagic degradation of glycogen (glycophagy) during fasting (Schiaffino and Hanzlíková, 1972; Jiang et al., 2010). Surprisingly, mice with constitutive hepatic mTORC1 activity by means of genetic deletion of *Tsc1* (Sengupta et al., 2010; Yecies et al., 2011; Liko et al., 2020), or liver-specific activation of RagA (de la Calle Arregui et al., 2021), do not phenocopy a deficiency in autophagy and instead have an impaired transcriptional adaptation to fasting that limits ketogenesis. Thus, in adult mice undergoing fasting, mTORC1 and autophagy control complementary adaptations to nutrient limitations (see Table 1).

### mTOR, Autophagy, and Lipid Homeostasis

In addition to supporting fasting glucose homeostasis, autophagy contributes to the hydrolysis of lipid droplets upon nutrient deprivation by the delivery of lipid droplets to lysosomes, and pharmacologic and genetic inhibition of autophagy in the liver leads to the accumulation of lipid droplets and to lower rates of β-oxidation both *in vitro* and *in vivo* (Singh et al., 2009a). *In vivo* deletion of *Atg7* specifically in WAT leads to a lean phenotype accompanied by morphological and functional changes of the WAT. Adipocytes from these animals are multilocular, with enriched content in mitochondria, and display increased rates of β-oxidation, and these mice also exhibit improved insulin sensitivity and resistance to high-fat-diet-induced obesity (Singh et al., 2009b; Zhang et al., 2009).

The phenotypes of mice with a hyper-active mTORC1 in the WAT have some features in common with those of defective autophagy. Deletion of *Tsc1* in adipocytes compromises WAT expansion, results in a higher mitochondrial oxidative activity and fatty acid oxidation and to a leaner phenotype when mice were fed a high-fat diet (Magdalon et al., 2016). While deletion of *Raptor* in WAT also results in defective WAT expansion, lipodystrophy, and resistance to diet-induced obesity (Lee et al., 2016), the overexpression of *Deptor*, a negative regulator of mTOR, promotes adipogenesis and mice gain more weight upon high-fat feeding (Laplante et al., 2012). *Deptor* overexpression decreases the negative feedback loop to Akt, while not significantly affecting other mTORC1 targets, supporting the notion that both mTORC1 and Akt activities are required for adipogenesis.

The mTORC1 target TFEB controls lipid catabolism at the transcriptional level by promoting PGC1-α-dependent PPARα activation in response to fasting (Settembre et al., 2013). An analogous process occurs in *C. elegans*, where HLH-30 (ortholog of TFEB) regulates the expression of lysosomal triglyceride lipases (O’Rourke and Ruvkun, 2013). Overexpression of *Tfeb* in mice (TFEB-Tg mice) leads to an improvement of glucose tolerance, insulin sensitivity, and weight gain in mice fed with high-fat diet and, importantly, to a lean phenotype that is abolished in an *Atg7*-deficient background, genetically demonstrating the epistasis of TFEB and autophagy (Settembre et al., 2013). This work is consistent with a report showing that autophagy is impaired in obese (ob/ob) mice, and reconstitution of autophagy in the liver by expressing *Atg7* using an adenoviral system improves their glucose tolerance and insulin sensitivity (Yang et al., 2010). Consistently, ubiquitous and moderate overexpression of *Atg5* in mice, resulting in increased autophagy, leads to a leaner phenotype, an increased glucose tolerance and insulin sensitivity, and resistance to age-induced obesity (Pyo et al., 2013).

In line with the phenotypes observed in TFEB-Tg mice and in *Atg5*-Tg mice, liver-specific *Raptor* KO mice are resistant to diet-induced obesity and to hepatic steatosis, and these effects are, at least in part, mediated by the mTORC1 target Lipin1 (Peterson et al., 2011). In an apparent contradiction, mice with hyperactive mTORC1 signaling in the liver (liver-specific *Tsc1* KO) are also resistant to age- and diet-induced hepatic steatosis (Sengupta et al., 2010), but this apparently counterintuitive finding can be explained by an indirect attenuation of Akt-SREBP1 signaling (rather by suppression of autophagy) through the induction of a negative feedback loop to the insulin receptor substrate (IRS) (Kenerson et al., 2011; Yecies et al., 2011).

Mouse genetics for the study of WAT can produce phenotypic findings of difficult interpretation. This difficulty is probably related to the strength of perturbation caused by genetic deletion, in contrast to more physiological activation or inhibition of a signaling pathway, and to the expression patterns and leakiness of Cre recombinases. Nonetheless, the *in vitro* and *in vivo* lessons together have taught us that an appropriate, dynamic, and physiological regulation of both mTORC1 activity and autophagy is necessary for adipogenesis and for maintaining the correct accumulation and mobilization of lipids and the physiological functioning of WAT (Clemente-Postigo et al., 2020) (Table 1).

### mTOR, Autophagy, and Skeletal Muscle Homeostasis

Another tissue that exhibits a remarkable sensitivity to changes in both autophagy and mTOR activity is skeletal muscle. The muscle has the intrinsic ability to hypertrophy and atrophy by a tight balance between protein synthesis and degradation (Fry and Rasmussen, 2011). Intense research has demonstrated that, although acute activation of the Akt–mTORC1 and the mTORC2–Akt axes drive hypertrophy, both chronically aberrant increase and decrease in mTOR activity yield muscle atrophy because dynamic synthesis–degradation oscillations are essential to maintain fiber homeostasis.

Muscle degradation occurs both in an autophagy-independent manner, by FoxO-dependent transactivation of the ubiquitin ligases Atrogin-1 and MuRF1 (Sandri et al., 2004; Stitt et al., 2004), and by autophagic degradation by FoxO-dependent transcription of many autophagy-related genes (Mammucari et al., 2007; Zhao et al., 2007). Thus, the Akt–FoxO signaling pathway coordinates the two main degradative pathways involved in atrophy: the ubiquitin–proteasome and the autophagy–lysosome.

In addition to a key role of the mTORC2–Akt–FoxO pathway in the control of muscle homeostasis, deletion of the mTORC1 component *Raptor* in the skeletal muscle results in profound atrophic and dystrophic muscles, with metabolic and structural changes incompatible with mouse survival (Bentzinger et al., 2008). While short-term genetic hyperactivation of mTORC1 in skeletal muscle by deletion of *Tsc1* results in hypertrophy, sustained mTORC1 hyperactivation eventually precipitates atrophy in most muscles and premature death (Bentzinger et al., 2013; Castets et al., 2013). The features of this myopathy are very similar to the ones developed by mice with a specific deletion of *Atg7* in the skeletal muscle, where aberrant membranous structures accumulate in muscle fibers (Masiero et al., 2009). The strong suppression of Akt through the mTORC1-mediated negative feedback loop allows the expression of Atrogin-1 and MuRF1 in young, but not in old, muscle-specific *Tsc1*-KO (sm-*Tsc1* KO) mice. Instead, the late-onset myopathy in skeletal muscle of old sm-*Tsc1* KO mice is caused by the inability to induce autophagy. Pharmacological inhibition of mTORC1 with rapamycin restores the autophagic flux and rescues the defects, pointing to pivotal and complex roles of both mTORC1 and TORC2 in the control of autophagy in the skeletal muscle from young and old individuals (Castets et al., 2013). Recent work has also shown that both the mTORC1–autophagy axis and mTORC1–Akt axis are critical for the coordination of recovery after denervation (Castets et al., 2019) (Table 1).

## Concluding Remarks

The multilayered functional interactions of the mTOR signaling pathways and the process of autophagy are deciphered to a great extent at the cellular level, but we still have an incomplete understanding of their integration at the physiopathological level. To what extent does a blockade in autophagy explain the consequences of deregulated mTOR activity? Would the sole inhibition of mTOR unleash physiological autophagy and its benefits? Mouse genetics has contributed substantial snapshots of phenotypic similarities, epistasis, and complementary metabolic functions of mTOR inhibition and autophagy in different organs and metabolic states. A challenge for the future is to translate this body of knowledge into significant support for pharmacological manipulation of mTOR and autophagy to improve metabolism and control the pathologies associated with nutrient overload.

## Author Contributions

ND-S and AE conceived, wrote, and edited the text and figures. Both authors contributed to the article and approved the submitted version.

## Conflict of Interest

The authors declare that the research was conducted in the absence of any commercial or financial relationships that could be construed as a potential conflict of interest.
